# An Integrated Tool to Study MHC Region: Accurate SNV Detection and HLA Genes Typing in Human MHC Region Using Targeted High-Throughput Sequencing

**DOI:** 10.1371/journal.pone.0069388

**Published:** 2013-07-24

**Authors:** Hongzhi Cao, Jinghua Wu, Yu Wang, Hui Jiang, Tao Zhang, Xiao Liu, Yinyin Xu, Dequan Liang, Peng Gao, Yepeng Sun, Benjamin Gifford, Mark D’Ascenzo, Xiaomin Liu, Laurent C. A. M. Tellier, Fang Yang, Xin Tong, Dan Chen, Jing Zheng, Weiyang Li, Todd Richmond, Xun Xu, Jun Wang, Yingrui Li

**Affiliations:** 1 Science and Technology Department, BGI-Shenzhen, Shenzhen, China; 2 Roche NimbleGen, Inc., Madison, Wisconsin, United States of America; 3 Department of Biology, University of Copenhagen, Copenhagen, Denmark; 4 King Abdulaziz University, Jeddah, Saudi Arabia; 5 The Novo Nordisk Foundation Center for Basic Metabolic Research, Faculty of Health Sciences, University of Copenhagen, Copenhagen, Denmark; Emory Univ. School of Medicine, United States of America

## Abstract

The major histocompatibility complex (MHC) is one of the most variable and gene-dense regions of the human genome. Most studies of the MHC, and associated regions, focus on minor variants and HLA typing, many of which have been demonstrated to be associated with human disease susceptibility and metabolic pathways. However, the detection of variants in the MHC region, and diagnostic HLA typing, still lacks a coherent, standardized, cost effective and high coverage protocol of clinical quality and reliability. In this paper, we presented such a method for the accurate detection of minor variants and HLA types in the human MHC region, using high-throughput, high-coverage sequencing of target regions. A probe set was designed to template upon the 8 annotated human MHC haplotypes, and to encompass the 5 megabases (Mb) of the extended MHC region. We deployed our probes upon three, genetically diverse human samples for probe set evaluation, and sequencing data show that ∼97% of the MHC region, and over 99% of the genes in MHC region, are covered with sufficient depth and good evenness. 98% of genotypes called by this capture sequencing prove consistent with established HapMap genotypes. We have concurrently developed a one-step pipeline for calling any HLA type referenced in the IMGT/HLA database from this target capture sequencing data, which shows over 96% typing accuracy when deployed at 4 digital resolution. This cost-effective and highly accurate approach for variant detection and HLA typing in the MHC region may lend further insight into immune-mediated diseases studies, and may find clinical utility in transplantation medicine research. This one-step pipeline is released for general evaluation and use by the scientific community.

## Introduction

The MHC region, one of the most gene-dense regions of the human genome, is located on the short arm of human chromosome 6. It covers over 200 genes, 128 of which are predicted to be expressed [1]. Most MHC genes play a fundamental role in immunity, and show a close relationship with immune-mediated diseases [2], [3]. Over 30 years, numerous studies have demonstrated association between some MHC alleles and some disease susceptibilities. Today, more than 100 diseases, - with relation to infection, inflammation, autoimmunity, drug sensitivity, and transplantation medicine -have been reported to be associated with proteins coded by the genes in MHC region [4].

In addition to its high gene density, the MHC region is also one of the most complex regions in the human genome, due to the extremely high density of polymorphism and linkage disequilibrium (LD). This inherent complexity has made identification of the underlying, causative variants contributing to disease phenotypes by genome-wide association study (GWAS) a challenge. The single nucleotide variations (SNV) which distinguish PGF and COX, the two most significant MHC haplotypes in the European population, are at variant densities of ∼3.4× 10^–3^, higher than the estimated average heterozygosity between any other two haplotypes in the human genome [5]. Recognizing the importance of fully informative polymorphism and haplotype maps of the MHC region, as pertaining to MHC-related-diseases, the MHC Haplotype Consortium has conducted the MHC Haplotype Project between 2000 and 2006, and provided the sequence and annotations of eight different HLA-homozygous-typing haplotypes (PGF, COX, QBL, APD, DBB, MANN, MCF and SSTO) [6].

The Single Nucleotide Polymorphism (SNP) count in dbSNP for the MHC region has increased rapidly from 69,076 SNPs in database version 130 (Apr 30, 2009) to 169,279 in database version 135 (Oct 13, 2011), due in large part to the progress of the 1000 genome project. Although next generation sequencing (NGS) has made the cost of human genome sequencing decrease dramatically, the detection and analysis of all variants in the human MHC region for a large human cohort by whole genome sequencing (WGS) is still a challenge for researchers. Particularly for researchers who focus on genomic variations in MHC region, and for researchers who use genomic information to do HLA typing, targeted region sequencing stands out as a promising and neotypal approach.

The human leukocyte antigen (HLA) genes, the most studied genes in the MHC region, encode cell-surface proteins responsible for antigen peptide presentation in adaptive immune response. Considering the key role of HLA genes in immunology, HLA typing is widely used for matching donors and recipients in organ and hematopoietic stem cell transplantation in the clinical context [7], [8], particularly for variants at the four gene loci HLA-A, -B, -C and -DRB1. High resolution HLA matching is required for unrelated donor hematopoietic cell transplants [9]. Although precise HLA matching improves overall transplant survival and reduces the risk of rejection, graft-versus-host disease (GVHD) remains a significant and potentially life-threatening complication after hematopoietic cell transplantation (HCT), even when using HLA-matched transplants. Besides the five classic HLA genes (HLA-A, -B, -C, -DRB1 and -DQB1), additional genes in the MHC region, encoding unidentified transplantation antigens, are proposed to be responsible for GVHD [10]. Pröll et al. found 3,025 small variations between recipients and donors undergoing unrelated HLA-matched allogeneic stem cell transplantation, and proposed that various differences causing non-synonymous amino acid exchanges may lead to GVHD [11]. Therefore, choosing an HLA matched unrelated donor with the smallest number of differences to the recipient may help to reduce risk of rejection and increase odds of transplantee survival.

Target region capture sequencing was developed mainly to enrich and sequence specific regions of particular interest, such as specific exons or entire exomes [12], [13]. Target Region Sequencing (TRS) may be a cost-effective solution for studying MHC region; however, successful enrichments of large, contiguous, highly variable genomic regions has previously been thought to be problematic, because of ubiquitous repetitive regions and otherwise high diversity between haplotypes. In this study, a probe set was carefully designed for the target regions, using sequences of eight MHC haplotypes, to capture DNA fragments from the human MHC region, and then processed via high throughput sequencing. Using this probe set, we are able to sequence ∼97% of the defined MHC region with high SNV calling accuracy by this capture sequencing technology. We have also developed a HLA typing pipeline to type all of the genes in the IMGT/HLA database (http://www.ebi.ac.uk/imgt/hla,Release 3.9.0) with high accuracy, using this capture sequencing data or whole genome sequencing data. This toolset will improve and extend the usage of both capture sequencing and whole genome sequencing data in related studies which rely on HLA typing.

## Results

### Evaluation of Target Capture Sequencing, in Terms of Coverage

We generate sequence libraries from genomic DNA (gDNA) of the three samples mentioned in the method. After filtering reads with low sequence quality or sequencing adaptor, the purged data are mapped to the human genome reference sequence *hg19,* and more than 60% of the mapped reads are proved to align to the MHC region (Table 1). Although the probes themselves cover only 72.8% of the defined MHC region (3,620,871 bp of 4,970,558 bp), the alignment result proves that 97% of the defined MHC region is covered by at least one read, and over 95% by at least four reads (Table 1).

**Table 1:** Data production and mapping results for the three samples used.

Samples	YH	NA18532	NA18555
Target region ( bp)	4970558	4970558	4970558
Raw reads	7988210	5377408	5457998
Raw data (Mb)	719	484	491
Mapped reads	7766430	4875514	4991960
Uniquely mapped reads	7317777	4559340	4672521
Reads uniquely mapped to target	4794113	3023307	3100770
Capture specificity	65.51%	66.31%	66.36%
Mean fold coverage depth (x)	87.32	55.09	56.50
Percent bases with coverage ≥1x	97.29	96.95	97.20
Percent bases with coverage ≥4x	96.52	95.66	95.95
Percent bases with coverage ≥10x	95.50	93.84	94.13

Target regions here refer to contiguous MHC regions, rather than to the region actually covered by the designed probes. Capture specificity is defined as the percentage of uniquely mapped reads aligning to the target region.

**Figure 1:**
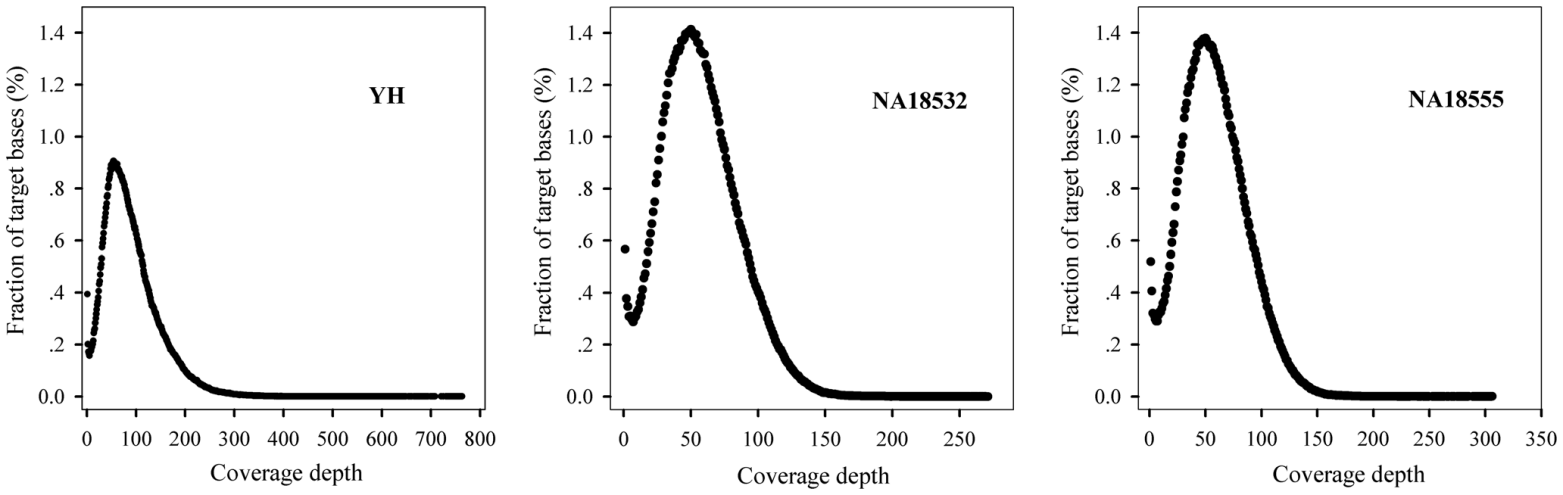
Distribution of per-base coverage depths in the MHC region for three samples. X-axis denotes coverage depth, Y-axis indicates percentage of total target region with a given sequencing depth. The fraction of target bases with zero coverage is not shown in the figure.

Several hundred samples have been captured and sequenced using this method, and the coverage of the MHC region is over 96% for all samples (data not shown). We investigated the 3–4% of uncovered regions and found that more than 99% of the uncovered bases were located in a long repeating region with length >2000 base per repeat (data not shown), which was difficult to solve with capture method due to NGS reads typically being too short to span the breadth of the long repeat region. The MHC region depth distribution showed a similar Poisson distribution for all three samples (Figure 1), indicating an even enrichment of the MHC region.

For further evaluation of the coverage of the genes in the MHC region, RefSeq genes are downloaded from the UCSC table browser (Hg19) [14], of which 213 genes and 380 transcripts are annotated in the defined MHC region. The average coverage of the longest transcripts of the 213 genes in the MHC region was 99.69% (Table S1). As to the coverage of the exons, the average coverage of all exons was 99.94% and 210 of 213 genes were 100% covered for all exons for all three samples (Table S1). This high coverage of the genes in MHC region makes it possible to detect all variations therein.

### Evaluation of Variation Detection Accuracy

Use of the capture sequencing method for mutation discovery is critically dependent on the accurate detection of polymorphisms and genotypes. Based on the target capture sequencing data, using GATK tools (v1.43) for YH, NA18532 and NA18555, respectively, we detected 19,002, 19,007 and 20,022 SNPs, and 2,332, 2,077, and 2,513 insertions and deletions (InDels).

**Figure 2:**
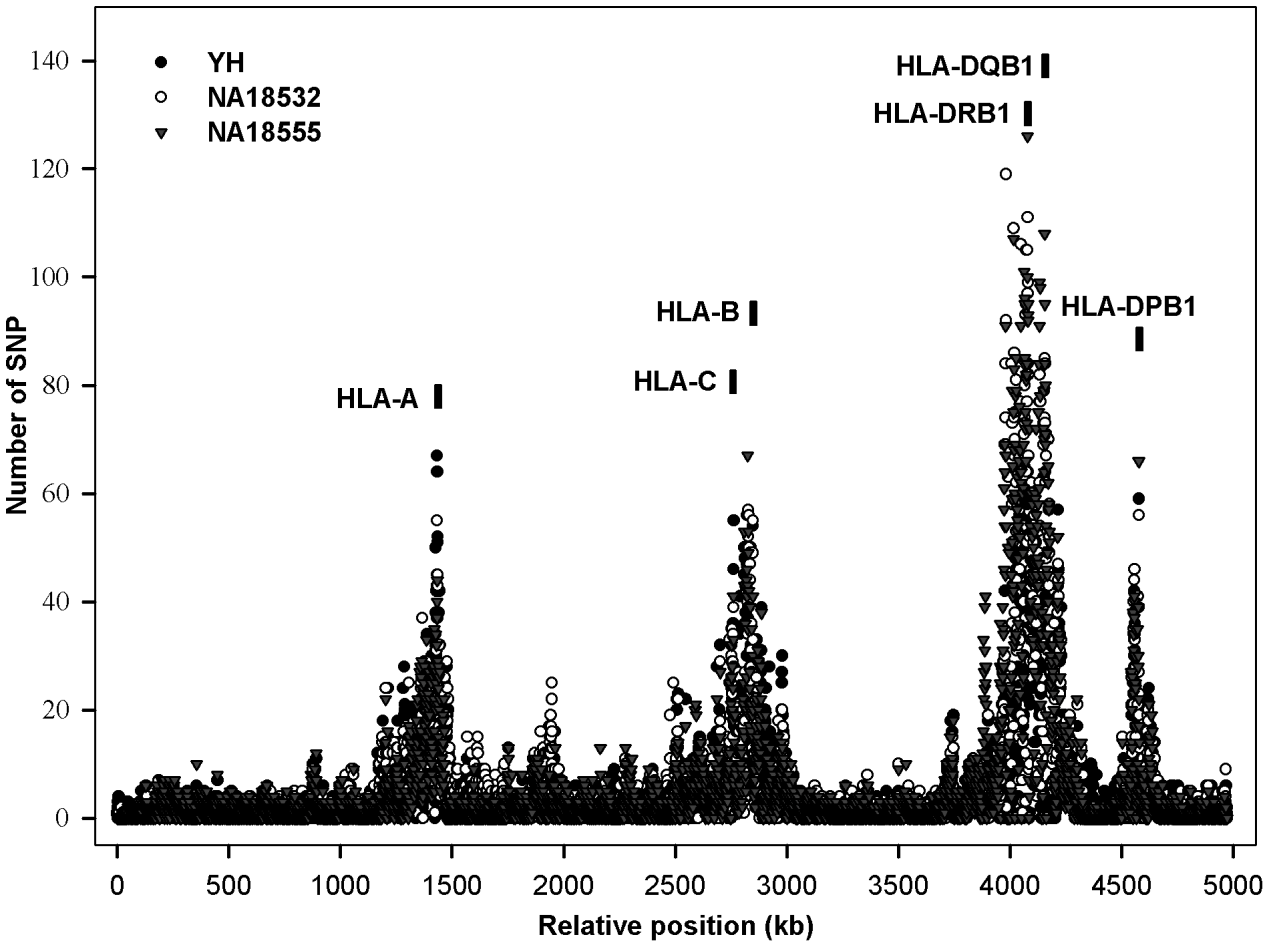
Single nucleotide variation distribution across the whole MHC region for three samples. The MHC region was split into 4971 parts, with 1000 bp in each part. X-axis denotes the 4971 parts and Y-axis indicates the number of SNPs in each part.

When analyzing the distribution of variations, we found that most of those SNPs were located in HLA-A, -B, -C, -DR, -DP and -DQ loci (Figure 2), leading to high degree of polymorphism within those genes. An average 3% of the SNPs identified in our study were determined to be novel, defined by their not being present in dbSNP132, or not being extant in the 1000 Genomes project (1000 Genomes Project Consortium, http://www.1000genomes.org, released on 23 November 2010). Using the ANNOVAR bioinformatics toolset (http://www.openbioinformatics.org/annovar) to functionally annotate the genetic variants in MHC region for the three case samples, we showed that about 2% of all detected variants were within exonic regions, 16% within intronic regions, and 70% within regions defined as intergenic (Table S2). Further investigation showed that most of the nonsynonymous variants resulting in changes to amino acid sequences - and hence probable functional differences in the protein - were also located in HLA-A, -B, -C, -DR, -DP and -DQ genes, putatively resulting in different peptide-binding interactions with T cell receptors.

**Figure 3:**
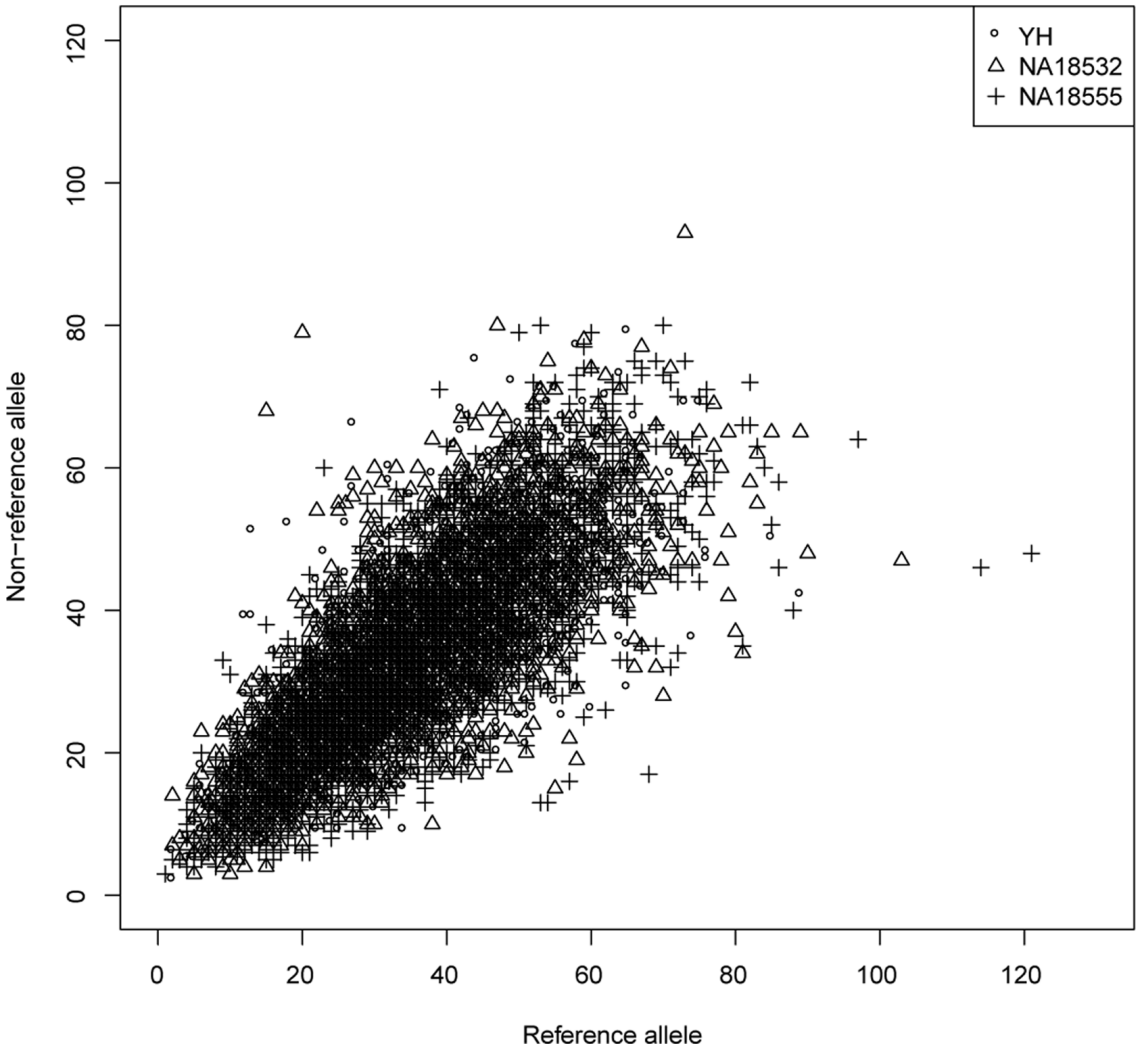
Capture bias evaluation using heterozygous genotypes in the Beadchip for three samples. X-axis denotes the support reads number of the reference allele and Y-axis denotes the support reads number of the non-reference allele.

In order to evaluate the accuracy of SNP detection, we compared our genotype result with the Illumina 2.5 M BeadChip genotype result for YH, and with HapMap genotype result for NA18555 and NA18532. There were 6,099 sites on the Illumina 2.5 M BeadChip which are within the MHC region, and 13,920 and 14,036 genotypes in the HapMap database for NA18532 and NA18555 respectively. 6,066 (99.5%), 13,775 (99.0%) and 13,895 (99.0%) of these genotypes are identified by target capture sequencing data with an accuracy of 98.83%, 97.82% and 98.26%, respectively, for the three study samples (Table S3, S4 and S5). The rate of divergence (about 2%) of our genotype results from the HapMap genotypes is calculated to be higher than the estimated average error rate of HapMap genotypes of 0.5% [15]. Two-thirds of the divergent loci show missing data at one allele in the HapMap genotype, which indicating that these inconsistent sites might result from failure of amplification, caused by the high background heterozygosity of the MHC region. Due to this high degree of divergence between any given pair of MHC haplotypes, biased enrichment data may likewise lead to an erroneous result. In order to evaluate the bias of our reads on two different MHC haplotypes, we therefore count the support read number for each allele at the heterozygous locus in the BeadChip for YH, and HapMap data for NA18532 and NA18555. This count shows that most of the sites had equal support reads for the non-reference allele and reference allele, by which we infer that our probes display good enrichment balance for the two haplotypes (Figure 3).

In order to validate the authenticity of the novel SNPs and InDels, two already proved fosmids covering the HLA–B gene locus were generated, based on our previous project using YH gDNA. Both of the selected fosmids covered the MHC region of chr6 ∶31,317,939 – 31,344,876 (hg19), but they each are templated on different haplotypes. Using the sequence data from fosmids, we validated the accuracy of 379 of the 382 SNPs (99.21%) (including 14 of the 15 novel SNPs) and 35 of the 37 InDels (94.59%) (data not shown).

### Description of HLA Typing Method

**Figure 4:**
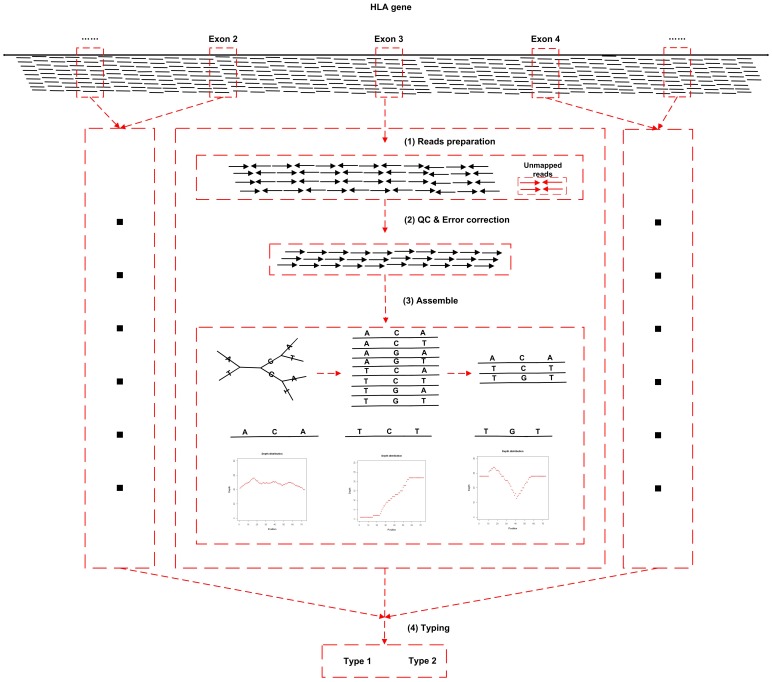
The workflow of HLA typing method.

Since the target capture sequencing data can be expected to cover more than 99% of genes in MHC region, it has the potential to give all MHC HLA gene types. Here, based on the above cited TRS data, we have developed an HLA typing pipeline which enables typing all of the HLA genes in the IMGT/HLA database until the date of publishing. The pipeline takes the aligned BAM files as input, and outputs the most reliable HLA types for each gene (Figure 4).

A brief summary of the pipeline was as follow:

(1) **Read preparation:** Paired reads with either end mapped to target gene region, and unmapped paired reads with high quality, are categorized by gene, and extracted from the BAM. (2) **Quality control and error correction:** The collected reads of each gene are aligned to the IMGT/HLA database, which contains the sequence of all currently known types, using Basic Local Alignment Search Tool (BLAST, version 2.2.24). Reads with a maximum of two discordant (SNP/InDel) to the most similar reference sequence in the database are kept and corrected to the closest reference sequence, while maintaining and adding to a record of the discordant SNP/InDel variants. (3) **Haplotype sequence assemble:** For each exon, reads are randomly assembled, with a minimum overlap region length of 10 bp for reads with no difference allowed in the overlap region, so as to construct haplotypes. Artificial haplotypes that do not exist in IMGT/HLA database are filtered out, and remaining haplotypes are given quality scores as defined below (see method). (4) **Typing according to assembled haplotype sequence**: The final haplotype of the sample is determined by rank of quality score (See method).

### Evaluation of HLA Typing Accuracy

Firstly, we test our HLA typing pipeline on simulated data. Based on the sequence of eight known haplotypes, sequenced by Sanger’s MHC haplotype project in 2008 with a foreknown HLA type result, we simulated regional sequence data for a diploid sample MHC, and ran the HLA typing pipeline upon it. For each sample, we typed 26 HLA genes, as recorded in IMGT/HLA database. The simulated data showed the accuracy of our method at 2 and 4 digital resolutions, and at sequencing depths ranging from 20× to 100× with sequencing error rates ranging from 0–2% (Table S6). The results of this typing were then validated against the HLA type given by the MHC haplotype project.

We then used this pipeline on three real human genome samples selected for this study. For each sample, we typed the same 26 HLA genes recorded in the IMGT/HLA database. In order to estimate the accuracy of the HLA typing result, we typed the nine HLA genes with the highest diversity in the IMGT/HLA database using the gold standard: Polymerase Chain Reaction (PCR) based Sanger sequencing, at 4 digital resolution. When our pipeline was compared with this standard, 100% of the 54 HLA alleles typed by our method were found consistent with those of the Sanger standard at 4 digital resolution (Table S7).

In a second test project, 190 human samples which had already been typed by traditional SSO or PCR based Sanger sequencing method (the same gold standard) were captured and sequenced using our pipeline, comparing in particular on five genes (HLA-A, -B, -C, -DRB1 and -DQB1). The comparison showed that the concordance of our method with the traditional gold standard was 96.4% at 4 digital resolution, and 98.8% at 2 digital resolution (data not shown). This result indicates the high performance and reliability of our targeted region pipeline.

## Discussion

In our study, we have developed an efficient approach for targeted region sequencing of the MHC region of the human genome, focusing on detection of minor variants and HLA types, in a high-coverage, high-reliability and low-cost framework. Beyond the 3.4 Mb of the standard MHC region, we designed probes for a continuous 5 Mb genomic region, to include further information from the extended high linkage disequilibrium area of the broader MHC region.

Our data presents 97% coverage for the broader MHC region and 99.69% for the transcripts of the crucial MHC genes, in all three testing samples. Compared with the method of Proll [11], our study showed a significantly higher coverage of the MHC region. Moreover, the accuracy of SNVs identified by our target capture sequencing data was in particularly high concordance with genotype data established by the most reliable methodology for this purpose, indicating the high degree of reliability of our approach.

To overcome the challenge of accurately genotyping the extremely high density of polymorphisms and linkage disequilibria in the human MHC region, our method optimized the standard target capture sequencing pipeline. Unlike traditional probes, which are designed only based on one of the haplotypes of the human reference sequence, the probe design for the MHC region in this study is based on all eight major MHC haplotype sequences. This enables capture of less common haplotypes in this region, ultimately reducing capture bias and failure. We also shear DNA fragments to ∼500 bp, increasing the coverage of the MHC region compared to the shorter fragments length around ∼200 bp (Table S8) which is more typically used in conventional exon capture libraries. This is particularly useful in the MHC because probes must be annealed to unique regions, but some repetitive regions, which are not covered by the probes, can be enriched by flanking to unique genome regions close to them, by using a longer and more unique genome fragment.

Based on this high-coverage, low-bias capture data, we achieve exceptionally accurate SNP and InDel results, and exceptionally accurate HLA gene type results in MHC region, at a level which before could only be achieved by a more specialized and expensive experimental method, or based on a large database. The traditionally utilized HLA typing methods - such as sequence specific oligonucleotide (SSO), sequence specific primer (SSP) [16], PCR based capillary sequencing and next generation sequencing method [17] - only detect variants in two or three exons, and it is very possible for new alleles in other regions of the gene to be missed when using these traditional methods. Another forwardlooking method has been reported for the imputation classical HLA alleles and corresponding amino acid based on the SNP information in the MHC region [18], but it demands a huge well studied reference panel. Our data covers essentially all regions of known HLA genes, and has the potential for more accuracy and higher resolution in typing, as well the potential for the detection of novel types (a not infrequent occurrence in this highly polymorphic region), and an important clinical clue in the event of tissue rejection and GVHD. High coverage data allows us to detect all variants and type all HLA genes in the MHC region in one-step with low cost. Some researchers suggest that the transplant barrier is defined not only by classical HLA genes, but also by non-HLA genetic variation within the MHC [10], but many previously dominant methods have been unable to gather data of sufficient breadth to verify or refute this contention.

One limitation of our target region method, as well as most other methods, is the inability to fully cover long repeated regions, and present 100% coverage of MHC region. This difficulty may later be overcome by shearing gDNA to longer fragments (5000 bp, or longer), capturing using probes of this size, and sequencing on the third generation sequencing platforms. A solution to this difficulty is important and may open the possibility of phasing long MHC haplotype blocks at low cost, ultimately reducing the risk of transplantation rejection and GVHD for those less able to afford a more expensive solution.

### Conclusion

The MHC region harbors the highest density of immunity-crucial genes in the human genome, and is arguably the region most central to immunogenetics and tissue transplantation. The ability to accurately call variants and HLA types is very important to immunology related studies in general, and to clinical application in particular. In this paper, we outline our development of a novel, low cost pipeline for the identification of variants and typing of HLA alleles in the MHC region in a one step protocol with high accuracy. This pipeline has great potential for use in the study of immune disease and transplantation medicine.

## Materials and Methods

### Design of MHC Capture Probes

The classical MHC region is defined as a ∼3.4 Mb region stretching between the gene C6orf40 (ZFP57) and the gene HCG24 [19]. In this study, due to extreme linkage disequilibrium of the region, and due to the presence of MHC-relevant genes nearby [19], we extended the MHC region to the telomeric gene GPX5 and the centromeric gene ZBTB9 in the probes design, corresponding to the genomic region from chr6∶28,477,797 to chr6∶33,448,354 in the human reference genome (NCBI release GRCh37, UCSC release hg19), encompassing a total of 4,970,558 bp.

Unlike previous studies of the MHC region, we also include the other seven MHC haplotypes aside from PGF - namely COX, QBL, APD, DBB, MANN, MCF and SSTO - in our probes design so as to improve the coverage of the MHC region and minimize the effect of the regionally high polymorphism density. A maximum allowance of greater than 5 close matches is set to get optimal capture efficiency of such high polymorphic regions. In total, 3,620,871 bp (about 72.8% of the 5 Mb MHC region) was covered by the capture probes for PGF, and the coverage rose to 4150331 bp (83.5%) when probes extended by 100 bp at both end. The design name is 110729_HG19_MHC_L2R_D03_EZ and design file can be downloaded from Roche NimbleGen website (http://www.nimblegen.com/products/seqcap/ez/designs/index.html) in the product “human MHC design”. Five capture arrays were initially produced for testing the uniformity of coverage, and the regions with extremely low or high sequencing depth were rebalanced by adjusting the number of probes - probe count for regions with extremely low sequencing depth was increased, and for regions with extremely high sequencing depth was decreased. Target region sequencing of the MHC was conducted for three samples using the set of rebalanced probes.

### Samples Collection

Three samples were used to evaluate the performance of the MHC region capture strategy. One was YH, which had previously been whole genome deep sequenced in 2008 [20], and genotyped using Illumina 2.5 M BeadChip. The other two samples were cell line DNA, NA18532 and NA18555, which were purchased from Coriell Institute, and which had previously been analyzed using high-throughput SNP genotyping in the HapMap project [21].

Nine HLA allele (HLA-A, HLA–B, HLA–C, HLA-DRB1, HLA-DQB1, HLA-DPA1, HLA-DPB1, HLA-DQA1, HLA-G) types for these samples were already detected using the gold standard sequence based typing method, in which the exonic regions of HLA genes were initially amplified by PCR, and the amplicons were subsequently sequenced using Sanger method.

### Whole Genome Shotgun Library Construction

High molecular weight gDNA was extracted using DNeasy Blood & Tissue Kits (QIAGEN, 69581). Shotgun libraries were generated from 3 micrograms ( µg) of genomic DNA followed by the manufacturer’s instruments (Illumina). gDNA in Tris-EDTA was sheared into 400–600 bp fragments using a Covaris S2 (Covaris). The overhangs were then converted to blunt ends, using T4 Polynucleotide Kinase, T4 DNA polymerase and Klenow polymerase. The fragments were then A-tailed using Klenow (3′–5′ exo-). Next, Illumina sequencing adapters with a single “T’ base overhang were linked to the A-tailed sample using T4 DNA Ligase. Finally, the fragments with adapters were enriched via four cycles of PCR.

### MHC Region Capture and High Throughput Sequencing

One µg of prepared sample library was hybridized to the capture probes following the manufacturer’s protocols (Roche NimbleGen). The hybridization mixture was incubated for 70 h at 65°C. After the hybridization was finished, the target fragments were captured with M-280 Streptavidin Dynabeads (Invitrogen), and then the captured sample was washed twice at 47°C and three more times at room temperature using the manufacturer’s buffers. The captured target fragments were amplified using Platinum® Pfx DNA Polymerase (Invitrogen) at the following condition: 94°C for 2 min, followed by 15 cycles of 94°C for 15 s, 58°C for 30 s, 72°C for 50 s and a final extension of 72°C for 5 min. The PCR products were purified and sequenced with standard 2× 91 paired-end reads on the Illumina HiSeq2000 sequencer following manufacturer’s instructions.

### Sequencing Read Mapping and Variant Calling

Low quality and adapter contaminated reads were filtered out to get the clean reads and then they were aligned to the reference assembly (UCSC Hg19) with haplotypes sequence removed using Burrows-Wheeler Aligner (BWA, version 0.5.9) with the parameters -o 1 -e 63 -i 15 -L -k 2 -l 31 -q 10–I [22]. PCR duplicates were removed by Samtools (version 0.1.17) [23]. Base qualities were recalibrated and reads were realigned around potential insertions and/or deletions using The Genome AnalysisToolkit (GATK) software (version 1.43). SNPs and InDel were also detected using GATK for the regions with at least 4× sequencing depth as described previously [24], [25].

### HLA Genes Haplotype Sequences Assemble

As showed in Figure 4, De novo assembling method was used to construct the haploid sequence for each exon by using overlapped reads. The assembled sequence was randomly paired if the whole exon was broken by low depth region. We filtered those sequences that did not exist in the IMGT/HLA database. As for the remaining haplotype sequences, we scored them according to the following formula:
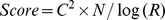



C: Coverage of this assembled sequence to its corresponding exon;

N: Number of reads used to construct the assembled sequence;

R: Reliability of this assembled sequence. It was calculated using the following formula:
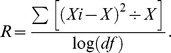



Xi: The depth of each base of the assembled sequence;

X: The mean depth of the assembled sequence;

df: The degrees of freedom of the assembled sequence. df = N−1, N equals the length of assembled sequence.

### Typing According to Assembled Haplotype Sequence

We combined the assembled sequences (called haplotypes) of all exons together to produce a group of types, and then scored them using the following formula:
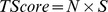



N: The number of reads that support the haplotype.

S: The score of the haplotype.

Based on the assumption that number of reads mapped to the correct haplotype is larger than that mapped to the incorrect haplotype, we first select the two types with the highest TScore, which can explain the highest number of the sequence reads for all exons as candidate types, and then we divide the sequence reads into two corresponding groups. We judge whether a HLA gene would be a homozygous or heterozygous, depending on the ratio of unique supporting reads number of the sub-optimal type, compared to the supporting reads number of the best type. The threshold of a heterozygote is 0.1. If the ratio is smaller then 0.1, the gene is defined as a homozygote, and the type with the largest score is chosen as the dominant type. Otherwise, the typed gene is defined as heterozygote, and the two types with the highest score are chosen as the final type result. Finally, we assess the reliability of the final type by comparing it with the closed type using AScore:
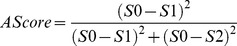



S0: The full score of the all assembled haplotypes of the typed gene;

S1: The score of the closed type;

S2: The score of the final type.

On the other hand, all the discordant information recorded in the process of ‘error correction’ was judged using variant calling methods to check whether there is a novel SNP or InDel comparing to an extant type. If so, the relationship of the novel variant to the given type was also described.

### Data Simulation and HLA Typing

Basing on the sequence of eight MHC haplotypes given by the MHC haplotype project in 2008, we randomly simulated one diploid sample’s MHC region sequence data using wgsim (v 0.2.3) provided by the Samtools package. First, we randomly selected two from the eight haploids, at various sequencing depths and various sequencing error rates using parameters –e and –N. The insertion size was set to 500 bp, which was the same as our target capture sequencing data, and the SD was 10. For each of the given conditions, we simulated 36 human MHC region data sets. We then worked out each simulated sample’s 26 HLA gene’s type result, using the method we describe above. The statistical accuracy result is given in Table S6.

### Data Access

The raw target capture sequencing data of MHC region for sample YH, NA18526 and NA18555 has been deposited at the NCBI Sequence Read Archive (SRA) under accession no. SRA065846. The HLA typing pipeline is implemented in Perl script and can be freely downloaded at http://soap.genomics.org.cn/SOAP-HLA.html.

## Supporting Information

Table S1 **Coverage of whole gene body and exome of the MHC genes by capture sequencing data.**
(XLSX)

Table S2 **Distribution of variations within different genomics functional regions for three samples.**
(XLSX)

Table S3 **Comparison of MHC capture SNPs and Illumina 2.5 M genotyping alleles for sample YH.** We classified both the MHC capture alleles and the alleles that were called by genotyping into three categories: (1) Hom ref (homozygotes where both alleles are identical to the reference); (2) Hom mut (homozygotes where both alleles differ from the reference); (3) Het ref (heterozygotes where only one allele is identical to the reference); The number of MHC capture sequencing sites that are consistent with genotyping at both alleles, at one allele, or that are inconsistent at both alleles were categorized as 2, 1, and 0, respectively.
(XLSX)

Table S4 **Comparison of MHC capture genotypes and HapMap genotypes for sample NA18532.**
(XLSX)

Table S5 **Comparison of MHC capture genotypes and HapMap genotypes for sample NA18555.**
(XLSX)

Table S6 **The accuracy of HLA typing method using simulated data at different sequencing depth with different sequencing error rate.**
(XLSX)

Table S7 **HLA alleles typed by PCR based Sanger sequence method and target capture sequence method.**
(XLSX)

Table S8 **Coverage of the MHC region using 200 bp insert size library.**
(XLSX)
